# Effect of integrated supportive supervision on availability of resources for health care service delivery and uptake of services in Ekiti State, Southwest Nigeria, evidence from the Saving One Million Lives program for result supported facilities

**DOI:** 10.11604/pamj.2024.47.45.34291

**Published:** 2024-02-06

**Authors:** Oluwafunmilayo Oluwadamilola Ibikunle, Ayobami Oyekunle Afape, Caroline Ajoke Bakare, Tope Michael Ipinnimo, Demilade Olusola Ibirongbe, Esther Opeyemi Ajidahun, Austine Idowu Ibikunle, Ayodele Gilbert Seluwa, Samuel Akinjide Akinleye, Oyebanji Filani

**Affiliations:** 1 Ekiti State Ministry of Health and Human Services, Ado-Ekiti, Nigeria,; 2 Heidelberg Institute of Global Health, Heidelberg, Germany,; 3 Ekiti State Primary Health Care Development Agency, Ado-Ekiti, Nigeria,; 4 Department of Community Medicine, Federal Teaching Hospital, Ido-Ekiti, Nigeria,; 5 Department of Community Medicine, University of Medical Sciences, Ondo, Nigeria,; 6 Department of Pediatrics, Wesley Guild Hospital Unit of Obafemi Awolowo University Teaching Hospital Complex, Ilesha, Nigeria

**Keywords:** Primary health care, integrated supportive supervision, Ekiti State

## Abstract

**Introduction:**

a world bank performance-based financing program. The Saving One Million Lives program for results supported integrated supportive supervision (ISS) in selected primary health facilities (PHF) in Ekiti State, Nigeria. The study assessed the impact of ISS on health service outputs and outcomes such as infrastructure, basic equipment, human resources for health (HRH), essential drugs, number of children receiving immunization, number of mothers who gave birth in the facility, number of new and continuing users of modern family planning and the number of pregnant women screened for HIV (human immunodeficiency virus).

**Methods:**

a cross-sectional survey of 70 SOME-supported facilities was used for the study. Parametric and non-parametric method of analysis was employed to compare the mean values of study indicators gathered over the 4 rounds of ISS visits from January 2018 to August 2020.

**Results:**

the study demonstrated that ISS approach has a positive effect on PHC service outputs and outcomes such as infrastructure, basic equipment, health human resources (HRH), essential drugs, contraceptives prevalence rate, skilled birth attendant as well as postnatal care. However, there was no significant impact on HIV screening for pregnant women.

**Conclusion:**

integrated supportive supervision approach has a positive effect on the quality of health care delivery in PHCs in Ekiti State, Nigeria. It is therefore recommended that periodic ISS visits should be routinely carried out in all PHCs across the State in the country and can be further extended to secondary and tertiary facilities.

## Introduction

Globally, improvements in community health are attributable to the provision of quality PHC services [1]. For a country to provide every citizen with equal rights to enjoy the highest attainable standard of health, the health system has to be built on the foundation of primary health care [2]. An efficient health care system is measured by the improved health outcomes of citizens by making quality services available to all through the bridging of the gap in the supply of quality PHC services [1,2]. Various measures have been proposed by the World Health Organization (WHO) towards ensuring the provision of quality PHC services at every level of the community to foster secure and equitable health services delivery [3]. According to the Donabedian model, in ensuring and assessing the quality of health care, health facilities must have structures (material resources, human resources, organizational structure), processes (what is done in giving and receiving care) and health outcomes [4]. In addition, supportive supervision is often considered an initiative that strengthens the health care system, allowing health care personnel to provide high-quality services, and enhances performance [5]. The embedment of supportive supervision within the health system structure facilitates an increase in the capacity of human resources for health by playing a crucial role as performance-enhancing techniques which contributes to quality improvement, especially in low-income settings [6]. Several studies have associated positive outcomes such as improved job performance, satisfaction and motivation with supportive supervision [7].

Additionally, support supervision also allows for team building, problem-solving, and regular feedback, which encourages skill development through training and mentorships [8,9]. However, despite the wide distribution of PHC centers across Nigeria, the performance of these health facilities in terms of service delivery has remained abysmally poor [10]. Factors such as inadequate supply chain, deplorable infrastructure state, inaccessibility to patients owing to financial constraints, poorly equipped states of the PHCs, and shortage of human resources and commodities (drugs, vaccines) have been implicated in the poor performance of PHCs [10]. As a result, the Nigerian health sector's performance on crucial indices has been ranked as one of the countries of the world with some of the poorest health indices [11]. Furthermore, there is still mixed and inconclusive evidence about the effectiveness of supportive supervision in low-income countries (Nigeria Inclusive) [12]. Some studies reveal that in many low-income countries (Nigeria inclusive) supervisory procedures are inadequate, coupled with a poorly skilled, insufficient and inefficiently managed health workforce [13,14]. While some studies showed that supportive supervision improved health care delivery outcomes at the PHC level [8,15-17]. In sub-Saharan Africa (Nigeria inclusive), there is a vast knowledge gap about the extent to which supportive supervision improves clinical outcomes [9] and also, less attention is being paid to initiatives that enhance the quality of care [18]. In Ekiti State, the Saving One Million Lives program for results (SOML PforR), a performance-based financing World Bank program, employed ISS as one of the key strategies aimed at driving institutional processes to achieve results by using technical officers to supervise activities to improve service delivery on maternal newborn and child health (MNCH) outcomes across health facilities in the State. This study aims to assess how ISS impacts the availability of resources and PHC services delivered in Ekiti State. The study measured the impact of ISS on health service outputs (structure) - infrastructure, basic equipment, human resources for health (HRH), essential drugs; and health outcomes (uptake of some services) - number of children receiving immunization, number of mothers who gave birth in the facility, number of new and continuing users of modern family planning and the number of pregnant women screened for HIV. Findings from this study would enhance the quality of ISS and evidence-based health care practices in Ekiti State and Nigeria.

## Methods

**Study design and setting:** the study was a cross-sectional survey carried out in Ekiti State. The State is located in Southwest Nigeria with a projected population of 3.4 million [19]. Ekiti State is made up of 16 Local Government Areas (LGAs) in three senatorial districts namely, Ekiti Central, Ekiti North and Ekiti South. The inhabitants of the State are mainly Yoruba speaking people and mostly Christians. The health system in the State consists of three tiers of health care (primary, secondary and tertiary) that are linked through referral to provide health services for the citizens. Currently, there are 326 PHC facilities, 22 general hospitals (3 specialist hospitals in each senatorial district inclusive with a minimum of 1 general hospital per LGAs) and 3 tertiary health facilities (1 state-owned, 1 federal-owned and 1 private-owned). Resources (personnel and funds) are distributed based on the level of health service provided.

**Data sources and measurement:** the SOML PforR is an ambitious effort to improve maternal and child health outcomes through strengthening existing health systems by non-state actors and government at all levels. The initiative focused on 6 key areas such as improving MNCH, improving coverage of routine immunization and eradicating polio, eliminating mother-to-child transmission of HIV, expanding access to essential medicines and commodities, combating malaria and improving child nutrition [20]. The intervention supported ISS to assess the implementation level of key health indices across 70 SOML supported health facilities in the State (a minimum of 4 primary health care centers were purposively selected in each LGA as they provide primary health care for 80% of the target population in each LGA).

The ISS approach under the SOML intervention consisted of three core elements. First, the team framework consisted of trained technical supervisors from the state and local levels. Second, the supervisory process involved a comprehensive supervisory visit that entailed a review of key indicators including on-the-job capacity building; discussion of key problems, action plans and agreement on following-up actions by both sides (supervisor and supervisees) within the agreed time frame. Lastly, the review mechanism comprised of feedback including a verbal and written report to the facility management at the local level and the State SOML secretariat respectively. Furthermore, a technical review meeting is conducted to aggregate the findings of the supervisory team and the performance of the health facilities in each region is discussed to inform the decision to improve the State health sector. The ISS data was collected from August 2018 to December 2019. The ISS checklist was used to assess the health facilities' service delivery performance supported by the SOML intervention [20]. The 70 SOML-supported PHC facilities from all the LGAs in the state formed the sample for the study. The facility ISS checklist was administered electronically by both state and LGA supervisory teams using the Survey CTO.

**Variables:** the study variables were availability of infrastructure and basic equipment, human resources and essential drugs, provision of immunization (Penta 1 and Penta 3), prenatal and postnatal care, family planning (contraceptive prevalence rate), skilled birth attendance and HIV screening. The questions that assessed these service delivery performance indicators were analyzed and expressed in percentages.

**Statistical methods:** the study indicators were analyzed and expressed in percentages. The mean values for each visit's indicators were computed and reported as mean and standard deviation for all of the study health facilities. Parametric conditions were explored before performing the analysis of variance (ANOVA) test. The conditions such as homogeneity of variances and normally distributed dependent variable were met by only four (4) variables: availability of human resources, immunization (pentavalent vaccine 1 and 3), and postnatal care. Thus, the effect of the four ISS visits on the study variables in the 70 health facilities was assessed using a one-way analysis of variance (ANOVA) and Tukey post hoc test. Other variables that did not meet all the parametric test assumptions such as infrastructure availability, basic equipment and essential drugs, postnatal care, skilled birth attendance, immunization, and HIV screening were analyzed using the Kruskal-Wallis H non-parametric test and Dunn post hoc. The data analysis was performed using the IBM SPPS for Window version 24.0 (IBM Corp., Armonk, N.Y, USA).

**Ethical consideration:** ethical approval for this study was gotten from Ethical Committee, Federal Teaching Hospital, Ido-Ekiti. Permission was also gotten from Ekiti State Ministry of Health and Human Services.

## Results

**Figure 1:**
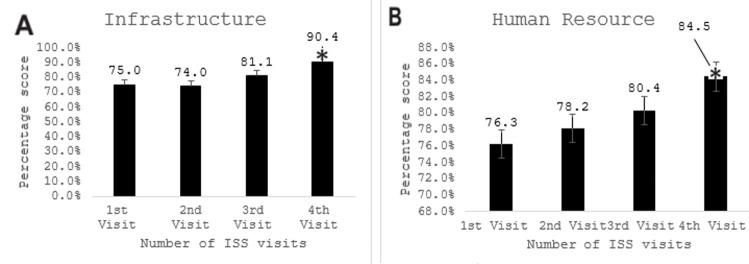
effect of integrated supportive supervision on infrastructure (A) and human resources (B)

**Figure 2:**
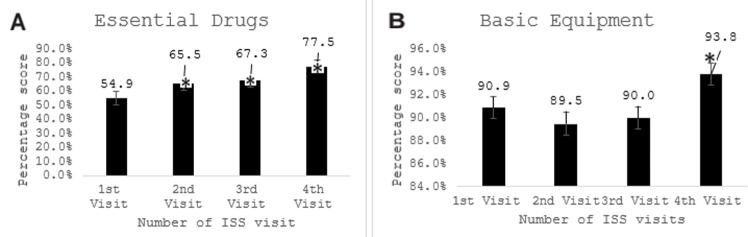
effect of integrated supportive supervision on essential drugs (A) and basic equipment (B)

**Figure 3:**
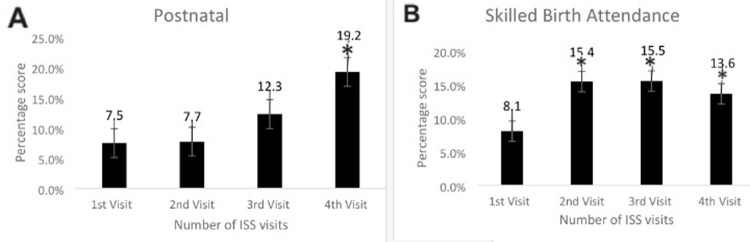
effect of integrated supportive supervision on postnatal (A) and skilled birth attendance (B)

**Figure 4:**
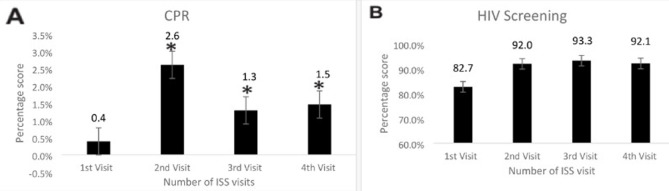
effect of integrated supportive supervision on contraceptive prevalence rate (A) and HIV screening (B)

The impact of the ISS visits to the 70 health care facilities was summarized in Table 1. Figure 1A showed that at the first ISS visit to the health facilities, the average availability of infrastructure was 75.00±18.55 (95% confidence interval 95% CI: 70.57%-79.42%). Except for a slight reduction in the average infrastructure score at the second visit, it increased steadily between the first and fourth visits; and was significant at the fourth visit (P <0.05). Figure 1B showed that HRH availability average at the first visit was 76.27±19.09 (95% CI: 71.73%-80.83%), this value increased with each ISS visit, but it was only significant at the fourth visit (P < 0.05). In Figure 2A, the findings revealed the essential drugs available in the health facilities steadily increased from an average of 54.92±21.87 (95% CI: 49.71%-60.14%) at the first ISS visit to an average of 77.54±13.75 (95% CI: 72.45%-81.12); which was significant for the fourth visit (P < 0.05). In Figure 2B, the average basic equipment available in the health facilities at the first visit was high (90.88±8.32; 95% CI: 88.89%-92.86%) and only increased significantly at the fourth ISS visit (P < 0.05). Figure 3A showed that the provision of postnatal care in the health facilities steadily increased from an average of 7.48±19.92 (95% CI: 2.73%-12.23%) at the first ISS visit, but the increase was only significant at the fourth visit (P < 0.05). As depicted in Figure 3B from the first visit, there was an increase in skilled birth attendance among pregnant women in the health facilities 8.05±9.13 (95% CI: 5.87%-10.23%). Compared to the first visit, there was a significant increase (P < .05) at the second, third and fourth visits. Also, Figure 4A showed the changes in the mean contraceptive prevalence rate (CPR) across the visits at the first visit 0.39±0.66 (95% CI: 0.23%-0.54%), compared to the first visit, there was a significant increase in the subsequent visits (P < 0.05). As shown in Figure 4B, compared to the first visit (82.72±33.40; 95% CI: 74.51%- 90.93%), the average number of pregnant women screened for HIV increased at the second, third and fourth visits but was not statistically significant (P >0.05).

**Table 1:** findings summary on the impact of integrated supportive supervision visit to 70 health care facilities in Ekiti State

Indicator	Score (%)			
	1^st^ Visit	2^nd^ Visit	3^rd^ Visit	4^th^ Visit
Infrastructure^a^	75.00 ± 18.55	74.00 ± 16.73	81.05 ± 14.23	90.37 ± 16.93*
Basic equipment^a^	90.88 ± 8.32	89.48 ± 8.45	89.97 ± 9.04	93.80 ± 6.27*
Human resource^b^	76.27 ± 9.09	78.18 ± 13.62	80.35 ± 14.01	84.52 ± 13.80*
Essential drug^a^	54.93 ± 21.88	65.54 ±18.35*	67.28 ± 20.00*	77.54 ± 13.75*
Penta 1	78.59 ± 27.75	66.99 ± 27.81	73.46 ± 25.72	74.07 ± 29.23
Penta 3	75.88 ± 28.47	65.24 ± 28.14	63.54 ± 26.86	64.30 ± 33.69
Prenatal	25.52 ± 24.99	24.84 ± 20.90	30.18 ± 28.20	26.70 ± 22.46
Postnatal^a^	7.48 ± 19.92	7.73 ± 17.99	12.26 ± 23.02	19.21 ± 24.55*
Skilled birth attendance^a^	8.05 ± 9.13	15.41 ± 15.41*	15.49 ± 14.71*	13.60 ± 13.27*
Contraceptive prevalence rate ^a^	0.39 ± 0.66	2.61 ± 6.30*	1.29 ± 1.67*	1.46 ± 1.96*
HIV screening^a^	82.72 ± 33.40	91.99 ± 24.80	93.28 ± 16.14	92.13 ± 22.84

a Asterisks (*) along rows indicates significantly different values from the 1^st^ visit (P < .05); the Kruskal-Wallis and Dunn post hoc test was used to establish the significance levels of the mean difference; ^b^Asterisks (*) along rows indicates significantly different values from the 1^st^ visit (P < .05). ANOVA followed by Tukey post hoc test was used to establish the significance level of the mean difference

## Discussion

Integrated supportive supervision is seen as a strategy that strengthens the health system, enables quality service delivery and improves performance [21]. This study assessed the impact of ISS on health care service outputs and outcomes in PHCs supported by the SOML intervention. The outcome parameters that were assessed included infrastructure, basic equipment, HRH, essential drugs, number of children receiving immunization, number of mothers who gave birth in the health facility, number of new and continuing users of modern family planning as well as the number of pregnant women screened for HIV. This study showed that ISS has a positive effect on the quality of health care service delivery, there was a significant improvement across almost all the outcome parameters except for immunization, prenatal care, and HIV screening across the four visits. This study showed that ISS has a positive effect on the availability of certain resources for health namely infrastructure, basic equipment, HRH, essential drugs; and uptake of certain services such as CPR, SBA delivery, and postnatal care. However, it had a significant improvement in uptake of immunization, prenatal care and HIV screening across the four visits. In this study, there was a significant improvement in infrastructure during the last visit. This result is similar to a study in Katsina State, Nigeria that found improvement in infrastructure after several ISS visits [8].

It is also similar to another study carried out in two States (Akwa Ibom and Zamfara States) in Nigeria that found an improvement in family planning infrastructure after a second ISS visit to the health facilities [22]. Also, there was an improvement in basic equipment following the fourth ISS visit. This finding is, however, different from findings in Nigeria and Tanzania where ISS did not have a significant impact on the availability of basic equipment [8,23]. In Africa, poor monitoring and supervision have been linked to a lack of basic medical equipment [24]. Furthermore, the boost in basic equipment available in the present study may be due to the procurement of essential life-saving commodities and equipment by the program to all the SOML-designated health facilities as well as by the state government and National Primary Health Care Development Agency (NPHCDA) to all PHCs in the state. The continuous ISS visits to SOML health facilities may likely help in maintaining and sustaining this procured equipment, although the non-SOML supported PHCs were not comparatively observed to see if the availability of this equipment differs.

There was a significant improvement in the essential drug's availability across the visits. This result is consistent with the result of a study that found an increase in essential drug availability in every subsequent visit [8]. However, this is in contrast with the findings of another study that found low availability of essential drugs [25]. The State Primary Health Care Development Agency implemented a policy that insisted that all PHCs must procure their drugs from a central medical store, as well as training officers in charge of drugs in PHCs on drug revolving policy. The continuous availability of essential drugs may be due to the fact that one of the focuses of the ISS was to remind the officers in charge of drugs of the important content of these drug revolving policies and guide them on proper implementation.

This study saw a significant improvement across all the visits in the area of skilled birth attendance and CPR. The significant increase in new family planning acceptors following ISS visits was consistent with the result of a previous study [22]. Also, SOML-supported interventions such as the engagement of skilled birth attendants with procurement and distribution of mama kits following the first ISS visit contributed to the improvement noticed in subsequent ISS visits to skilled birth attendance. There was also a significant improvement in HRH, this is at variance with a previous study in which no significant impact of ISS on the number of health care workers was found [8]. However, a systemic review done in sub-Saharan Africa showed that supportive supervision can increase job satisfaction as well as health care worker motivation [5]. Job satisfaction and motivation may serve as pull factors in addition to retaining health care workers. Moreover, high-quality supportive supervision can improve health service delivery among delinquent health workers in changing their negative attitudes and reaching their full potential, as well as people achieving optimal health [26].

HIV screening in this study showed no significant improvement. This result is not similar to that in Katsina State, Nigeria, which showed a significant increase in the number of pregnant women screened for HIV [8]. The low HIV coverage in this study may be due to the initial absence of HIV test kits in the state. Also, the steady increase in HIV coverage though not statistically significant may be due to the procurement of HIV test kits through the support of the SOML program. Immunization coverage did not show significant improvement in all the ISS visits. This is consistent with findings in other studies carried out in Nigeria and Zambia [8,27]. The finding may be due to the fact that strategies to improve immunization coverage go beyond the health facilities; it includes other stakeholders such as the caregivers and the community.

**Limitations:** this study used a cross-sectional study design, which cannot give a causal relationship. We recommend that subsequent studies on the effect of ISS on health care service delivery should employ a more robust design that would provide a direct causal association between parameters of interest.

## Conclusion

This study demonstrated that ISS approach has a positive effect on the availability of certain resources and uptake of certain services needed in health care delivery through PHCs in Ekiti State, Nigeria. The key parameters improved by ISS in this study were infrastructure, basic equipment, HRH, essential drugs, CPR, skilled birth attendant as well as postnatal care. It is therefore recommended that periodic ISS visits should be routinely carried out in all PHCs across the state. The State Ministry of Health, WHO and other partners should provide more backing for ISS in terms of funds, logistics and materials for significant impact on primary health care delivery

### What is known about this topic

- *Poor quality of health care service delivery still persists in many low- and middle-income countries;*
- *In many resource-limited settings, there is still mixed and inconclusive evidence about the effectiveness of supportive supervision*.

### What this study adds

- *Integrated supportive supervision has positive effect on key primary health care service delivery parameters;*
- *Consistent use of ISS improved key parameters in the areas of infrastructure, basic equipment, HRH, essential drugs, contraceptive prevalence rate skilled birth attendant and postnatal care*.

## References

[1] Kruk ME, Porignon D, Rockers PC, Van Lerberghe W (2010). The contribution of primary care to health and health systems in low-and middle-income countries: a critical review of major primary care initiatives. Soc Sci Med.

[2] Friedberg MW, Hussey PS, Schneider EC (2010). Primary care: a critical review of the evidence on quality and costs of health care. Health Aff (Millwood).

[3] World Health Organization (2008). The world health report 2008: primary health care now more than ever. World Health Organization.

[4] Donabedian A (1988). The quality of care. How can it be assessed?. JAMA.

[5] Bailey C, Blake C, Schriver M, Cubaka VK, Thomas T, Martin Hilber A (2016). A systematic review of supportive supervision as a strategy to improve primary healthcare services in sub-Saharan Africa. Int J Gynaecol Obstet.

[6] Flahault D, Piot M, Franklin A, World Health Organization (1988). The supervision of health personnel at district level.

[7] Bradley S, Kamwendo F, Masanja H, Pinho H, Waxman R, Boostrom C (2013). District Health managers´ perceptions of supervision in Malawi and Tanzania. Hum Resour Health.

[8] Nass SS, Isah MB, Sani A (2019). Effect of Integrated Supportive Supervision on the Quality of Health-Care Service Delivery in Katsina State, Northwest Nigeria. Health Serv Res Manag Epidemiol.

[9] Bradley J, Igras S (2005). Improving the quality of child health services: participatory action by providers. Int J Qual Health Care.

[10] Kress D, Su Y, Wang H (2016). Assessment of Primary Health Care System Performance in Nigeria: Using the Primary Health Care Performance Indicator Conceptual Framework. Health Systems and Reform.

[11] Global Resource Center (2011). Country Coordination and Facilitation (CCF): Principles and Process.

[12] Glenton C, Colvin CJ, Carlsen B, Swartz A, Lewin S, Noyes J (2013). Barriers and facilitators to the implementation of lay health worker programmes to improve access to maternal and child health: qualitative evidence synthesis. Cochrane Database Syst Rev.

[13] Willcox ML, Peersman W, Daou P, Diakité C, Bajunirwe F, Mubangizi V (2015). Human resources for primary health care in sub-Saharan Africa: progress or stagnation?. Hum Resour Health.

[14] Manongi RN, Marchant TC, Bygbjerg IC (2006). Improving motivation among primary health care workers in Tanzania: a health worker perspective. Hum Resour Health.

[15] Purity M, Eilish M, Ogenna U, Honorati M, Henry M (2017). The impact of supportive supervision on the implementation of HRM processes; a mixed-methods study in Tanzania. Health Syst Policy Res.

[16] Djibuti M, Gotsadze G, Zoidze A, Mataradze G, Esmail LC, Kohler JC (2009). The role of supportive supervision on immunization program outcome-a randomized field trial from Georgia. BMC Int Health Hum Rights.

[17] Marquez LR, Kean LC Making supervision supportive and sustainable: new approaches to old problems.

[18] Alenoghena I, Alphonsus OA, Chukwuyem A, Eboreime E (2014). Primary health care in Nigeria: Strategies and constraints in implementation. IJCR.

[19] National Population Commission Population and Housing Census 2006.

[20] Federal Ministry of Health (2016). Saving one million lives program for results program implementation manual (PIM).

[21] Avortri GS, Nabukalu JB, Nabyonga-Orem J (2019). Supportive supervision to improve service delivery in low-income countries: is there a conceptual problem or a strategy problem. BMJ Glob Health.

[22] Adetiloye O (2017). The use of integrated supportive supervision (ISS) visits to strengthen family planning service delivery in two selected states of Nigeria. TIJPH.

[23] Mboya D, Mshana C, Kessy F, Alba S, Lengeler C, Renggli S (2016). Embedding systematic quality assessments in supportive supervision at primary healthcare level: application of an electronic Tool to Improve Quality of Healthcare in Tanzania. BMC Health Serv Res.

[24] Moyimane MB, Matlala SF, Kekana MP (2017). Experiences of nurses on the critical shortage of medical equipment at a rural district hospital in South Africa: a qualitative study. Pan Afr Med J.

[25] Oyekale AS (2017). Assessment of primary health care facilities´ service readiness in Nigeria. BMC Health Serv Res.

[26] Anoke C, Onuoha H, Njoku Chukwu E, Aka E, Ukemezia P (2021). Effects of supportive supervision on improved quality healthcare service delivery.

[27] Umar AS, Bello IM, Okeibunor JC, Mkanda P, Akpan GU, Manyanya D (2021). The Effect of Real Time Integrated Supportive Supervision Visits on the Performance of Health Workers in Zambia. J Immunol Sci.

